# Social Determinants of Emergency Department Utilization, Overuse, and Access Barriers Among Adults: A Systematic Review

**DOI:** 10.7759/cureus.110248

**Published:** 2026-06-04

**Authors:** Thouraya Elawad, Reem E Elshaikh, Mohamed Hamid, Fatima Ali, Hisham E Elsheikh, Taqwa E Elshaikh, Asim Ahmed

**Affiliations:** 1 Department of Emergency Medicine, Johns Hopkins Aramco Healthcare, Al-Mubarraz, SAU; 2 Department of Emergency Medicine, Dr. Sulaiman Al Habib Hospital, Riyadh, SAU; 3 Medicine and Surgery, University of Gezira, Wad Madani, SDN

**Keywords:** access barriers, barriers to access, emergency department overuse, health equity, non-urgent emergency department attendance, primary care access

## Abstract

Emergency departments (EDs) are facing increasing adult demand, and socially driven patterns of repeated, avoidable, or nonurgent use remain insufficiently synthesized across healthcare settings. ED utilization is influenced not only by clinical need but also by broader social and structural conditions that shape access to healthcare. This systematic review examined the social determinants associated with ED utilization, overuse, repeated attendance, and access barriers among adults. A structured search of major biomedical and health services databases was conducted, and eligible studies were reviewed using predefined inclusion and exclusion criteria. Because of variation across study designs, populations, and outcome definitions, findings were synthesized narratively. The review found that ED use was consistently associated with socioeconomic disadvantage, unmet social needs, poor access to timely primary care, weak social support, neighborhood deprivation, and broader health system barriers. Common drivers included low income, food insecurity, public insurance dependence, unstable housing, limited continuity of care, transportation difficulties, and restricted availability of alternative services. Across settings, repeated or nonurgent ED use often appeared to reflect unmet need and constrained access rather than simple inappropriate use. These findings suggest that socially driven ED utilization should be understood as a health equity issue requiring more responsive primary care, stronger social support pathways, and better integration between emergency services and community-based care.

## Introduction and background

Emergency departments (EDs) are a core component of modern health systems, providing immediate, unscheduled care for acute illness and injury [1]. However, emergency care systems worldwide continue to face persistent pressure. Crowding, delayed throughput, and rising demand cause important consequences for quality, safety, and efficiency [2-4]. Frequent ED users account for a disproportionate share of visits. Previous reviews show that this group often has complex clinical and service-use profiles, rather than simply reflecting inappropriate attendance [5-7]. In this review, ED overuse refers broadly to repeated or potentially preventable use; avoidable use refers to visits that may have been managed in a more appropriate alternative care setting; and non-urgent attendance refers to presentations that do not require immediate emergency intervention, while recognizing that these definitions vary across studies and health systems.

There is increasing recognition that ED utilization is shaped not only by clinical need but also by the social determinants of health. Socioeconomic disadvantage, limited access to routine care, unstable social conditions, and broader structural inequities can affect healthcare access and ED use patterns [8,9]. Research on non-urgent ED attendance shows visits are often driven by perceived urgency, difficulty obtaining timely primary care, convenience, advice from others, and the absence of acceptable alternatives. These are not simply patient preferences alone [10]. ED utilization also varies according to primary care access, older age, and geographic accessibility. This includes travel distance and the availability of alternative services [11-13].

Although the literature recognizes the role of social and structural determinants in emergency care, evidence remains fragmented. Most reviews focus on specific subgroups or dimensions of ED use. They rarely synthesize how social disadvantage, unmet social needs, access barriers, and health system gaps shape adult ED use across settings [5,8,10,12,13]. How these factors interact to drive repeated attendance, overuse, and non-urgent reliance on EDs is unclear. This systematic review examined which social determinants are associated with ED use, overuse, repeated attendance, and access barriers among adults. Specifically, it identified the social determinants most associated with use and overuse, determined the most common factors behind frequent or repeated visits, examined access barriers influencing attendance, assessed disparities among populations, and identified gaps for future research, policy, and interventions.

## Review

Methods

Study Design and Reporting Framework

This study was a systematic review with a narrative synthesis. It examined how social determinants influence ED use, overuse, repeat visits, and access barriers among adults. Reporting followed the Preferred Reporting Items for Systematic reviews and Meta-Analyses (PRISMA) 2020 Statement [14]. This review was not prospectively registered. Meta-analysis was not performed. Substantial heterogeneity was anticipated across study designs, populations, ED definitions, and outcome reporting. The review question was: Which social determinants are associated with ED use, overuse, and access barriers among adults?

Objectives

The primary objective was to identify the social determinants associated with ED utilization and overuse among adults. Secondary objectives were to determine the most commonly reported social determinants linked to frequent or repeated use; examine access barriers contributing to attendance; assess disparities across population groups; and identify implications for future research, policy, and service delivery. A Population, Intervention, Comparison, and Outcome (PICO)-informed framework guided the development of the eligibility criteria and data extraction (Table 1).

**Table 1:** PICO framework for the review question PICO, Population, Exposure or Interest (Intervention), Comparison, Outcomes.

Component	Definition
Population	Adults aged 18 years and older
Exposure or interest	Social determinants of health and structural barriers, including income, poverty, education, employment, insurance status, housing instability, transportation barriers, access to primary care, language or immigration factors, social support, and neighborhood deprivation
Comparison	Adults without the determinant of interest, or comparisons across exposure levels where applicable; qualitative studies did not require a formal comparator
Outcomes	Emergency department utilization, frequent or repeated use, non-urgent attendance, avoidable use, revisits, emergency department use as a usual source of care, and barriers to accessing alternative care

Information Sources and Search Strategy

PubMed/MEDLINE (Medical Literature Analysis and Retrieval System Online), Scopus, Web of Science Core Collection, and Cumulative Index to Nursing and Allied Health Literature (CINAHL) were searched. Backward citation screening was also performed. The search combined ED care, social determinants or access barriers, and utilization outcomes. Broad terminology was used to capture variations in reporting, including ED use, attendance, frequent use, non-urgent use, avoidable use, and revisits. Filters for adults and the English language were applied where available. Database-specific search strategies are in Table 2.

**Table 2:** Database search strategies ED, emergency department; Mesh, Medical Subject Headings; tiab, title and abstract; TS, topic search

Database	Search string
PubMed or MEDLINE	((“Emergency Service, Hospital”[Mesh] OR “emergency department*”[tiab] OR “emergency room*”[tiab] OR ED[tiab]) AND (“Social Determinants of Health”[Mesh] OR “social determinant*”[tiab] OR socioeconomic[tiab] OR income[tiab] OR poverty[tiab] OR education[tiab] OR employment[tiab] OR insurance[tiab] OR uninsured[tiab] OR housing[tiab] OR homeless*[tiab] OR transportation[tiab] OR “access to care”[tiab] OR “primary care”[tiab] OR language[tiab] OR immigration[tiab] OR migrant*[tiab] OR “social support”[tiab] OR deprivation[tiab] OR neighborhood[tiab] OR neighbourhood[tiab]) AND (utilization[tiab] OR use[tiab] OR attendance[tiab] OR visit*[tiab] OR overuse[tiab] OR “frequent use”[tiab] OR “repeated visit*”[tiab] OR revisit*[tiab] OR “non-urgent”[tiab] OR avoidable[tiab])) AND (adult[Mesh] OR adult*[tiab])
Scopus	TITLE-ABS-KEY((“emergency department*” OR “emergency room*” OR ED) AND (“social determinant*” OR socioeconomic OR income OR poverty OR education OR employment OR insurance OR housing OR homeless* OR transportation OR “access to care” OR “primary care” OR language OR immigration OR migrant* OR “social support” OR deprivation OR neighborhood OR neighbourhood) AND (utilization OR use OR attendance OR visit* OR overuse OR “frequent use” OR “repeated visit*” OR revisit* OR “non-urgent” OR avoidable) AND adult*)
Web of Science Core Collection	TS=((“emergency department*” OR “emergency room*” OR ED) AND (“social determinant*” OR socioeconomic OR income OR poverty OR education OR employment OR insurance OR housing OR homeless* OR transportation OR “access to care” OR “primary care” OR language OR immigration OR migrant* OR “social support” OR deprivation OR neighborhood OR neighbourhood) AND (utilization OR use OR attendance OR visit* OR overuse OR “frequent use” OR “repeated visit*” OR revisit* OR “non-urgent” OR avoidable) AND adult*)
CINAHL	((“emergency department*” OR “emergency room*” OR ED) AND (“social determinant*” OR socioeconomic OR income OR poverty OR education OR employment OR insurance OR housing OR homelessness OR transportation OR “access to care” OR “primary care” OR language OR immigration OR “social support” OR deprivation OR neighborhood) AND (utilization OR attendance OR visit* OR overuse OR “frequent use” OR revisit* OR “non-urgent” OR avoidable) AND adult*)

Eligibility Criteria

Studies were included if they examined adults aged 18 years and older and evaluated social determinants of ED utilization, frequent use, non-urgent use, overuse, or barriers to accessing care in ED settings. Eligible study designs included observational, qualitative, mixed-methods, and health services research. Included articles had to report outcomes on attendance patterns, repeated use, access barriers, or disparities, and be full-text, peer-reviewed publications.

Studies were excluded if they focused only on pediatric or adolescent populations, examined only clinical or disease-specific factors without social determinants, concentrated solely on trauma, disaster, or ambulance logistics, or were case reports, editorials, commentaries, conference abstracts, letters, or non-systematic reviews. Exclusions also included studies conducted outside emergency care settings, non-English articles, and those without an accessible full text.

Study Selection

All records were de-duplicated before screening. Titles and abstracts were screened against predefined eligibility criteria. Potentially relevant studies were then assessed for full text. Records were excluded if they were duplicates, non-original publications, conference abstracts, not focused on adult emergency care, or did not evaluate social determinants of ED use. The selection process followed PRISMA 2020 recommendations [14].

Data Extraction and Synthesis

A standardized approach was used to extract study characteristics, including author and year, country and setting, study design, population characteristics, ED outcomes, social determinants, access barriers, disparity-related findings, and key conclusions. Terms such as avoidable, non-urgent, and frequent ED use were extracted according to the operational definitions used in each original study and were not reclassified by the review team. Due to heterogeneity, findings were synthesized narratively rather than quantitatively. The synthesis was organized by determinant domains, patterns of ED use, access barriers, and disparities across population groups.

Risk of Bias Assessment

Methodological quality and risk of bias were assessed at the individual study level using Joanna Briggs Institute (JBI) critical appraisal tools selected according to study design, in line with JBI guidance [15,16]. The JBI Checklist for Analytical Cross-Sectional Studies was used for cross-sectional, survey-based, retrospective, population-based, and administrative quantitative studies without longitudinal follow-up. The JBI Checklist for Cohort Studies was used for cohort and linked observational studies. The JBI Checklist for Qualitative Research was used for qualitative studies. Studies were grouped by design before appraisal to support a consistent interpretation of methodological limitations. 

Results

Study Selection

**Figure 1:**
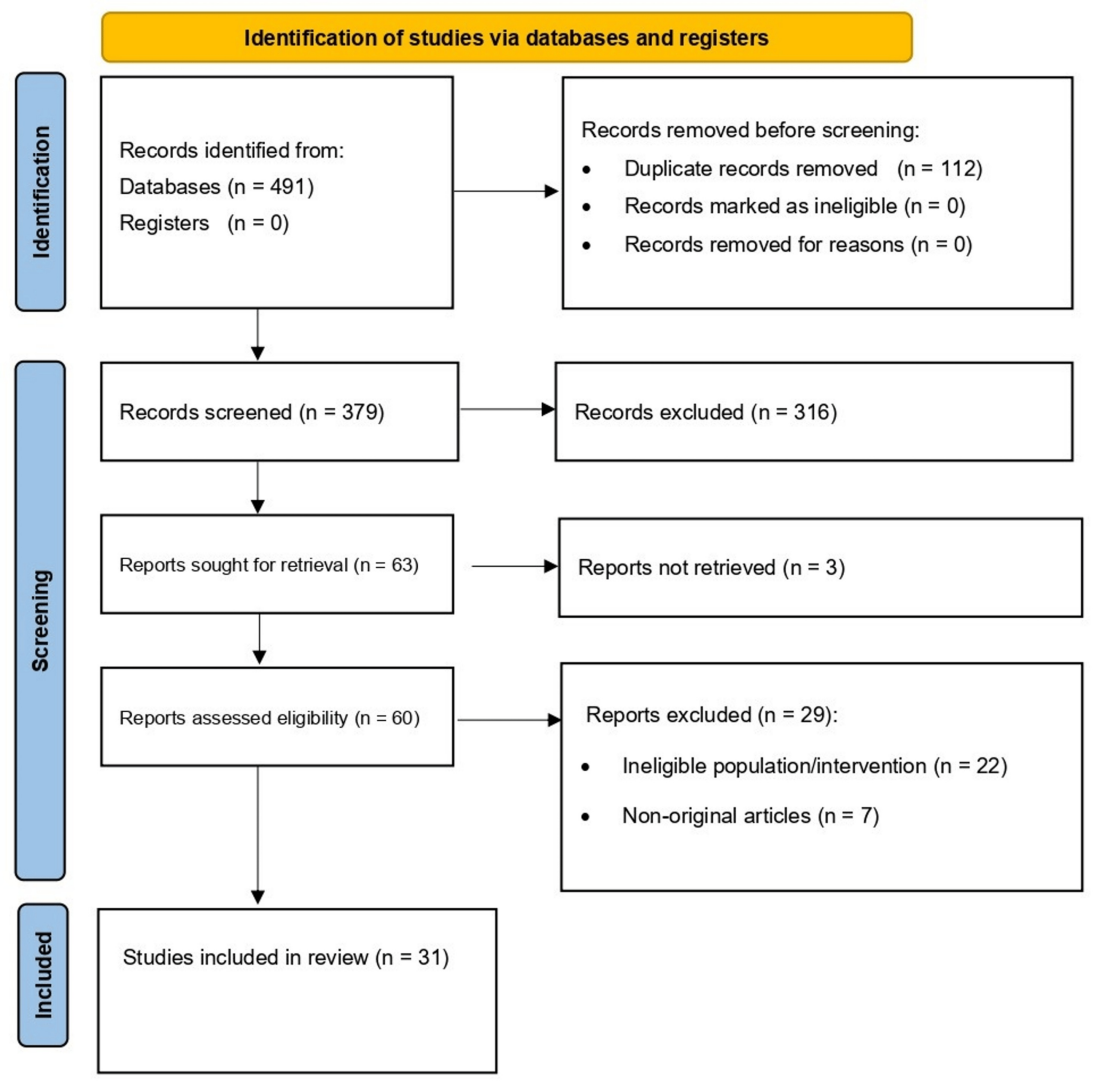
PRISMA 2020 flow diagram of study selection. PRISMA, Preferred Reporting Items for Systematic Reviews and Meta-Analyses.

A total of 491 records were identified through database searching. After removal of 112 duplicate records, 379 records remained for title and abstract screening. Of these, 316 records were excluded. Sixty-three full-text reports were sought for retrieval, and three could not be retrieved. Therefore, 60 full-text articles were assessed for eligibility. After full-text review, 29 articles were excluded because of ineligible population or setting, insufficient focus on social determinants of ED utilization, non-original publication type, overlapping data, or bibliographic ineligibility. A total of 31 studies were included in the narrative synthesis (Figure 1).

Overview of Included Studies

The final evidence base included population-based surveys, cohort studies, retrospective database studies, cross-sectional analyses, and qualitative studies conducted across North America, Europe, the Middle East, Africa, and Asia. The included studies examined overall ED attendance, frequent or repeated use, avoidable or non-urgent use, ED use as a usual source of care, and potentially preventable utilization. Across the included literature, the main determinants studied were poverty, low income, material deprivation, food insecurity, housing instability, low educational attainment, unemployment, public insurance, weak social support, barriers to primary care, rurality, neighborhood deprivation, and language- or immigration-related barriers [17-47]. Table 3 gives the characteristics of the included studies.

**Table 3:** Characteristics of included studies (N = 31) ED, emergency department.

Study (author, year)	Country and setting	Design	Main emergency department outcome	Main extracted determinant(s) or barrier(s)
Thompson et al., 2025 [17]	Ontario, Canada	Population-based descriptive study	Frequent, avoidable emergency department use	Low income, rurality, material deprivation, poor access to usual provider
Hunt et al., 2006 [18]	United States	Cross-sectional survey	Frequent emergency department use	Social disadvantage, poor physical and mental health, higher care burden
Lim et al., 2024 [19]	United States	Cross-sectional National Health Interview Survey study	One or more emergency department visits	Poverty, low education, unemployment, unmarried status, racial disparities
Da’ar et al., 2019 [20]	Riyadh, Saudi Arabia	Cross-sectional study	Frequency of emergency department use	Low income, low education, prior hospitalization, social reinforcement factors
Parashar et al., 2014 [21]	Canada	Cohort study	Emergency department use and repeated or non-urgent use	Unstable housing
Fleury et al., 2019 [22]	Quebec, Canada	Observational study	Low, moderate, and high emergency department use	Social and material deprivation, mental and physical illness complexity
Krebs et al., 2017 [23]	Canada	Observational emergency department study	Low-acuity emergency department use and avoidability	Family physician connection, marital status, employment, race
Cheung et al., 2012 [24]	United States	National survey study	Emergency department utilization	Barriers to timely primary care
Guleria et al., 2025 [25]	United States	National cross-sectional study	Any emergency department use	Economic instability, food insecurity, social isolation, educational deficits
Abbott et al., 2025 [26]	United States	Retrospective survey-weighted cohort	Repeated emergency department use	Unmet health-related social needs, food insecurity, delayed care due to cost, access difficulty
Hong et al., 2007 [27]	United States	Urban emergency department survey	Emergency department as routine healthcare source	Race or ethnicity, socioeconomic status, insurance, perceptions of care
Shippee et al., 2014 [28]	United States	Observational linked survey-administrative study	Emergency department as usual care source and repeated visits	Financial and non-financial barriers
Unger et al., 2022 [29]	Jerusalem, Israel	Cross-sectional questionnaire study	Repeat emergency department visits	Poverty, safety concerns, language barriers, knowledge of rights
Dufour et al., 2020 [30]	Quebec, Canada	Population-based cohort study	Frequent emergency department use	Rurality, social deprivation, material deprivation
Behr et al., 2016 [31]	United States	Cross-sectional survey	Frequent non-emergent emergency department use	Predisposing, enabling, social network, and service quality factors
Matifary et al., 2021 [32]	Kenya	Qualitative study	Non-urgent emergency department attendance	Primary care barriers, convenience, comprehensive emergency department care
Chandekar et al., 2026 [33]	United States	Cross-sectional study with logistic regression	Non-urgent emergency department visits	Low primary care visit frequency, public insurance
Afilalo et al., 2004 [34]	Canada	Comparative cross-sectional study	Non-urgent emergency department attendance	Barriers to primary care, accessibility, trust, referral
Chary et al., 2025 [35]	United States	Qualitative study	Frequent emergency department use	Social needs, health-status complexity, access barriers
Cho et al., 2023 [36]	South Korea	Nationwide cross-sectional study	Frequent emergency department use	Medical Aid, low income, regional resource imbalance
Zuckerman et al., 2004 [37]	United States	National cross-sectional study	Occasional and frequent emergency department use	Insurance coverage, access to care, health status
Petersen et al., 1998 [38]	United States	Cross-sectional study	Non-urgent emergency department use	No regular doctor
Sieck et al., 2016 [39]	United States	Qualitative interviews	Non-urgent emergency department use	Transportation, appointment barriers, awareness of alternatives
Durand et al., 2012 [40]	France	Qualitative interview study	Non-urgent emergency department attendance	Primary care physician access difficulty, convenience, one-stop diagnostics
Rudge et al., 2013 [41]	England	Retrospective database study	Emergency department attendance rate	Neighborhood deprivation, distance, proximity of alternative care
Carlson et al., 2021 [42]	United States	Area-level retrospective analysis	Potentially preventable emergency department use	Neighborhood socioeconomic risk
Ohle et al., 2018 [43]	Canada	Population cross-sectional study	Emergency department as regular care access point	Immigration status, education, employment, health status
Butler et al., 1998 [44]	United States	Observational study	Non-emergency emergency department use	Managed care and sociodemographic access factors
McKenna et al., 2020 [45]	United Kingdom	Qualitative network-mapping study	Avoidable or non-urgent emergency department attendance	Social networks, lack of mobilizable support, professional influencers
Rust et al., 2008 [46]	United States	Cross-sectional study	Any emergency department visit	Practical barriers to timely primary care
Brown et al., 1994 [47]	Ontario, Canada	Population-based survey	Any emergency department use and visit count	Health needs, sociodemographic factors, access-related influences

Social Determinants Associated with ED Utilization and Overuse

ED utilization and overuse were consistently associated with socioeconomic disadvantage. The most recurrent determinants were income-related disadvantage, material deprivation, low educational attainment, unemployment, weak social support, and poor continuity of primary care. Thompson et al. reported that frequent users with potentially avoidable visits were concentrated in the lowest income groups, rural settings, and materially deprived neighborhoods [17]. Lim et al. showed that among older adults with multimorbidity, low education, poverty, unemployment, and unmarried status were associated with one or more ED visits [19]. Abbott et al. found that unmet health-related social needs, particularly food insecurity, delayed care due to cost, and difficulty accessing medical care, increased the likelihood of repeated ED use [26]. Similar associations involving low income, deprivation, social vulnerability, housing instability, and insurance-related disadvantage were reported across multiple studies [20-22,28-31,35-37,41-47].

Most Commonly Reported Social Determinants Linked to Frequent or Repeated ED Use

Among studies specifically examining frequent or repeated ED use, the most consistent determinants clustered around economic and material hardship, limited social support, unmet social needs, and weak continuity of primary care. Guleria et al. identified economic instability, lack of community support, educational deficits, food insecurity, and social isolation as important drivers of ED use in the general adult population [25]. Abbott et al. found that the presence of at least one unmet health-related social need increased the odds of two or more ED visits within one year [26]. Thompson et al. reported that frequent avoidable ED users clustered in the lowest income quintiles and in materially deprived and rural areas [17]. Comparable findings were also observed among older adults, individuals with mental health vulnerability, and those experiencing unstable housing [19,21,22,30,35].

Access Barriers Contributing to ED Attendance

Access barriers extended beyond insurance status alone. Common barriers included delayed care due to cost, poor appointment availability, lack of a regular doctor, transportation difficulties, limited clinic hours, childcare constraints, and the perception that EDs provide more comprehensive or efficient care than primary care services. Cheung et al. [24] and Shippee et al. [28] showed that among publicly insured populations, non-financial barriers often predicted ED use more strongly than financial barriers alone. Qualitative studies indicated that patients frequently chose EDs because of one-stop diagnostics, rapid assessment, and greater confidence in care [32,39,40,45]. Geographic barriers, including rurality, travel distance, and limited availability of alternative services, also contributed to utilization patterns [17,30,41,46].

Disparities in ED Utilization Across Population Groups

Disparities in ED utilization were observed across age, ethnicity, immigration status, geography, and socioeconomic position. Lim et al. found higher ED use among older adults with multimorbidity [19], while Abbott et al. reported repeated ED use among Medicare beneficiaries with unmet health-related social needs [26]. Ethnic and racial disparities were identified in national and local studies, including higher ED use among non-Hispanic Black older adults and lower use among non-Hispanic Asian older adults compared with NHW older adults, as well as differences in repeat use between Arab and Jewish patients in Jerusalem [19,29]. Rural populations and individuals living in highly deprived areas were more likely to experience frequent or avoidable ED use [17,30,41]. Immigration status also influenced utilization patterns; Ohle et al. found that immigrants without a primary care physician were less likely than non-immigrants to use EDs as a regular source of care [43].

Narrative Synthesis and Implications for Research, Policy, and Service Delivery

Overall, the evidence indicates that ED overuse is influenced not only by clinical need but also by social vulnerability, limited access to timely primary care, fragmented support systems, and structural inequities. Across studies, nominal access to care, such as insurance coverage or a usual provider, did not consistently prevent repeated ED use among socially or medically complex populations [18,19,23,28,47]. Key gaps included inconsistent definitions of frequent or non-urgent use, heterogeneity in outcome reporting, limited longitudinal evidence, and a lack of intervention studies integrating social and healthcare services. These findings highlight the need for improved integration of social risk assessment, enhanced primary care access, and stronger coordination between emergency services and community-based support systems [17,25,26,45].

Risk of Bias Assessment

Overall, the risk of bias across the included studies was variable and closely related to study design, sampling methods, outcome definition, and control of confounding. Most quantitative studies were observational, cross-sectional, retrospective, or survey-based, which limited causal inference and increased susceptibility to selection bias, recall bias, misclassification, and residual confounding. Studies based on large national, administrative, or population-level datasets were methodologically stronger because they used larger samples, clearly defined ED outcomes, and multivariable analyses, although they remained limited by the observational nature of the data and by the possibility of incomplete measurement of social determinants [17,19,25,26,30,36,41-43,47]. Single-center and survey-based studies were generally more vulnerable to self-report bias, restricted generalizability, and incomplete adjustment for clinical and social confounders [18,20,23,24,29,31,33,34,37,38,44,46]. The qualitative studies contributed important contextual insight into access barriers, patient reasoning, and system influences, but their findings were inherently limited by purposive sampling, smaller sample sizes, and reduced transferability beyond the study settings [32,35,39,40,45]. No study was excluded on the basis of methodological quality, and all findings were interpreted in light of design-specific limitations. For a summary of the methodological strengths, main sources of bias, and overall risk-of-bias interpretation by study design, see Table 4.

**Table 4:** Summary of risk of bias across included studies by study design Risk of bias was interpreted according to study design, sampling strategy, measurement of social determinants and emergency department outcomes, degree of adjustment for confounding, and completeness of reporting. Lower to moderate risk of bias indicates stronger methodological structure but persistent limitations typical of observational research. Moderate risk of bias indicates meaningful design or reporting limitations that may affect the strength or generalizability of associations. No study was excluded on the basis of methodological quality.

Study design	Included studies (author, year)	Main methodological strengths	Main sources of bias or limitation	Overall risk-of-bias interpretation
Population-based, national, and administrative quantitative studies	Thompson et al., 2025 [17]; Lim et al., 2024 [19]; Guleria et al., 2025 [25]; Abbott et al., 2025 [26]; Dufour et al., 2020 [30]; Cho et al., 2023 [36]; Rudge et al., 2013 [41]; Carlson et al., 2021 [42]; Ohle et al., 2018 [43]; Brown et al., 1994 [47]	Large samples, broader population coverage, structured datasets, and use of regression or adjusted analyses	Observational design, possible misclassification of exposure or outcome, limited capture of social complexity, and residual confounding	Lower to moderate risk of bias
Cohort and linked observational studies	Parashar et al., 2014 [21]; Fleury et al., 2019 [22]; Shippee et al., 2014 [28]	Ability to examine repeated use or utilization patterns with more structured outcome assessment	Confounding, possible selection bias, limited causal inference, and dependence on available recorded variables	Moderate risk of bias
Cross-sectional and survey-based quantitative studies	Hunt et al., 2006 [18]; Da’ar et al., 2019 [20]; Krebs et al., 2017 [23]; Cheung et al., 2012 [24]; Hong et al., 2007 [27]; Unger et al., 2022 [29]; Behr et al., 2016 [31]; Chandekar et al., 2026 [33]; Afilalo et al., 2004 [34]; Zuckerman et al., 2004 [37]; Petersen et al., 1998 [38]; Butler et al., 1998 [44]; Rust et al., 2008 [46]	Useful for identifying associations between social determinants and emergency department use, often with direct patient-reported data	Selection bias, recall bias, self-report bias, single-center recruitment in some studies, and limited temporal inference	Moderate risk of bias
Qualitative studies	Matifary et al., 2021 [32]; Chary et al., 2025 [35]; Sieck et al., 2016 [39]; Durand et al., 2012 [40]; McKenna et al., 2020 [45]	Rich contextual data on barriers, perceived urgency, convenience, navigation, and social influence	Small purposive samples, limited transferability, possible interviewer and interpretive bias, and absence of quantitative effect estimates	Moderate risk of bias for qualitative inquiry, with limited generalizability

Discussion

Principal Findings

This systematic review showed that ED utilization, including frequent, repeated, and non-urgent use, was consistently associated with socioeconomic disadvantage, unmet social needs, weak primary care access, and broader health system barriers [17,19,20,23-26,28,36-41,45-47]. Across the included studies, the most recurrent determinants were low income, poverty, public insurance coverage, food insecurity, low educational attainment, neighborhood deprivation, weak social support, and practical barriers to timely primary care [17,19,20,24-26,28,29,36,37,41,45-47]. Poor primary care access emerged as a persistent system-level driver, including lack of a regular doctor, limited appointment availability, restricted clinic hours, transportation difficulties, childcare constraints, and poor continuity of care [23,24,28,34,38-40,45,46].

Poverty, Unmet Social Needs, and ED Reliance

Poverty and unmet social needs appeared to increase ED utilization by limiting access to routine care and reducing patients’ capacity for effective self-management. Abbott et al. showed that food insecurity, delayed care due to cost, and difficulty accessing care were associated with repeated ED use [26]. Guleria et al. found that food insecurity, economic instability, and social isolation were independently associated with ED use [25]. Lim et al. showed that among older adults with multimorbidity, poverty, unemployment, and unmarried status were associated with increased ED use [19]. Thompson et al. reported that frequent avoidable users were concentrated in the lowest income groups and materially deprived neighborhoods [17]. Da’ar et al. found that lower income and lower education were associated with higher visit frequency, while social support reduced attendance [20]. Together, these findings indicate that social disadvantage contributes to delayed, unstable, and unsupported disease management.

Primary Care Access as a System-Level Driver

Poor primary care access was a central driver of frequent and non-urgent ED use. Krebs et al. found that many low-acuity presentations were potentially avoidable with improved primary care access and continuity [23]. Cheung et al. demonstrated that barriers to timely primary care were strongly associated with ED use [24]. Shippee et al. showed that non-financial barriers, including childcare, transportation, and limited clinic hours, were more strongly associated with repeated visits than financial barriers alone [28]. Petersen et al. reported that the absence of a regular doctor increased the likelihood of non-urgent use [38]. Qualitative studies further showed that transportation barriers, limited appointment availability, low awareness of alternatives, convenience, and access to diagnostics influenced patient decisions to attend EDs [32,39,40,45]. These findings indicate that fragmented outpatient care contributes directly to ED reliance.

Frequent ED Use is Not Simply Inappropriate Use

Frequent or repeated ED use should not be interpreted solely as inappropriate utilization. Rather, the included studies suggest that repeated use often reflects unmet medical and social needs within constrained care systems. Abbott et al. showed that repeated ED use was associated with unmet health-related social needs among Medicare beneficiaries [26]. Lim et al. showed that ED use among older adults with multimorbidity was associated with social disadvantage [19]. Thompson et al. found that frequent avoidable use was concentrated in low-income and materially deprived neighborhoods, supporting the interpretation that repeated attendance often reflects social vulnerability rather than simple misuse [17]. Durand et al. described non-urgent users as rational decision-makers selecting accessible and comprehensive care [40]. McKenna et al. showed that some ED visits were influenced by wider personal and professional networks [45]. Studies such as those by Cho et al. [36] and Zuckerman et al. [37] further indicated that frequent users represent a clinically and socially complex population. These findings support interpreting repeated use as a marker of unmet need rather than misuse.

International Patterns and Context-Specific differences

Although the overall pattern was broadly consistent across countries, the dominant determinants varied by context and health system structure. In the United States, food insecurity, public insurance, unmet social needs, and access barriers were prominent [24-26,37-39,46]. In Canada, rurality, continuity of care, housing instability, and avoidable use were more frequently reported [17,21,23,30,43,47]. Cho et al. highlighted the role of Medical Aid and regional imbalance in South Korea [36]. Rudge et al. showed strong effects of deprivation and distance in England [41]. Unger et al. reported variation by ethnic group in Jerusalem [29]. Durand et al. highlighted convenience and access factors in France [40]. These findings indicate that while determinants are broadly consistent, their expression depends on healthcare structure and social context.

Disparities Across Population Groups

ED utilization varied across age, ethnicity, geography, insurance status, and housing conditions. Lim et al. [19] and Abbott et al. [26] reported higher utilization among older adults and older beneficiary populations. Ethnic and racial disparities were identified across multiple studies [19,27,29]. Thompson et al. [17] and Rudge et al. [41] highlighted the combined effects of deprivation and geography. Parashar et al. [21] identified housing instability as a key determinant. Ohle et al. found lower use among immigrants despite limited primary care access [43]. These findings indicate that ED overuse is concentrated among populations with overlapping vulnerabilities.

Implications for Practice and Policy

The findings support the integration of social risk screening into emergency care, including assessment of food insecurity, transportation barriers, and housing instability [21,25,26]. Improving primary care access requires timely appointments, continuity, extended hours, and practical support for social constraints [23,24,28,38-40,46]. Integration of social support services, care navigation, and community referral pathways may reduce avoidable use, particularly among high-risk groups [19,20,25,26,35,45]. Policy responses should address geographic and resource disparities, especially in underserved and rural areas [17,36,41].

Strengths and Limitations

This review included studies from multiple healthcare systems and populations and incorporated both quantitative and qualitative evidence [19,21,22,24,26,28-30,35,36]. However, most studies were cross-sectional or retrospective, limiting causal inference [17,19,24,25,28,36,37,41,42,46,47]. Definitions of frequent and non-urgent use varied across studies, reducing comparability [17,31,36,38,40]. Social determinants were measured inconsistently, and residual confounding may be present [25,26,28]. Restriction to English-language full-text studies may have excluded relevant evidence. Heterogeneity in design and outcomes precluded meta-analysis.

Future Research Directions

Future research should examine causal pathways, cumulative social risk, and intervention effectiveness. Longitudinal studies are needed to assess progression to repeated ED use. Standardized definitions would improve comparability. Intervention and mixed-methods studies are needed to evaluate whether addressing social needs reduces ED utilization and improves equity across settings.

Overall Interpretation

Overall, repeated and potentially avoidable ED use appears to reflect unmet social needs, limited access to routine care, and fragmented health systems rather than simple misuse alone. Repeated use is concentrated among individuals with combined clinical and social vulnerability. Addressing this issue requires coordinated improvements in primary care access, social support integration, and system-level responsiveness.

## Conclusions

ED overuse and repeated attendance among adults are strongly associated with social disadvantage, unmet needs, and barriers to accessible primary care. ED reliance often reflects responses to limited alternatives and fragmented systems rather than inappropriate patient behavior. Reducing avoidable ED use requires timely and continuous primary care, improved access to social support, and stronger coordination between emergency services and community-based care.

## References

[1] Morley C, Unwin M, Peterson GM, Stankovich J, Kinsman L (2018). Emergency department crowding: a systematic review of causes, consequences and solutions. PLoS One.

[2] Pearce S, Marchand T, Shannon T, Ganshorn H, Lang E (2023). Emergency department crowding: an overview of reviews describing measures causes, and harms. Intern Emerg Med.

[3] Hoot NR, Aronsky D (2008). Systematic review of emergency department crowding: causes, effects, and solutions. Ann Emerg Med.

[4] Savioli G, Ceresa IF, Gri N (2022). Emergency department overcrowding: understanding the factors to find corresponding solutions. J Pers Med.

[5] Soril LJ, Leggett LE, Lorenzetti DL, Noseworthy TW, Clement FM (2016). Characteristics of frequent users of the emergency department in the general adult population: a systematic review of international healthcare systems. Health Policy.

[6] van Tiel S, Rood PP, Bertoli-Avella AM (2015). Systematic review of frequent users of emergency departments in non-US hospitals: state of the art. Eur J Emerg Med.

[7] Althaus F, Paroz S, Hugli O, Ghali WA, Daeppen JB, Peytremann-Bridevaux I, Bodenmann P (2011). Effectiveness of interventions targeting frequent users of emergency departments: a systematic review. Ann Emerg Med.

[8] Morisod K, Luta X, Marti J, Spycher J, Malebranche M, Bodenmann P (2021). Measuring health equity in emergency care using routinely collected data: a systematic review. Health Equity.

[9] Davis CI, Montgomery AE, Dichter ME, Taylor LD, Blosnich JR (2020). Social determinants and emergency department utilization: findings from the Veterans Health Administration. Am J Emerg Med.

[10] McIntyre A, Janzen S, Shepherd L, Kerr M, Booth R (2023). An integrative review of adult patient-reported reasons for non-urgent use of the emergency department. BMC Nurs.

[11] Oterino de la Fuente D, Baños Pino JF, Blanco VF, Alvarez AR (2007). Does better access to primary care reduce utilization of hospital accident and emergency departments? A time-series analysis. Eur J Public Health.

[12] McCusker J, Karp I, Cardin S (2003). Determinants of emergency department visits by older adults: a systematic review. Acad Emerg Med.

[13] Kelekar U, Das Gupta D, Theis-Mahon N, Fashingbauer E, Huang B (2024). Distances to emergency departments and non-urgent utilization of medical services: a systematic review. Glob Health Action.

[14] Page MJ, McKenzie JE, Bossuyt PM (2021). The PRISMA 2020 statement: an updated guideline for reporting systematic reviews. BMJ.

[15] Joanna Briggs Institute (2026). JBI: Critical Appraisal Tools. Joanna Briggs Institute.

[16] Aromataris E, Lockwood C, Porritt K, Pilla B, Jordan Z (2024). JBI Manual for Evidence Synthesis.

[17] Thompson C, Watson T, Schull MJ, Gronsbell J, Rosella LC (2025). Sociodemographic and health behaviour of frequent, avoidable emergency department users in Ontario, Canada: a population-based descriptive study. West J Emerg Med.

[18] Hunt KA, Weber EJ, Showstack JA, Colby DC, Callaham ML (2006). Characteristics of frequent users of emergency departments. Ann Emerg Med.

[19] Lim A, Benjasirisan C, Liu X, Ogungbe O, Himmelfarb CD, Davidson P, Koirala B (2024). Social determinants of health and emergency department visits among older adults with multimorbidity: insight from 2010 to 2018 National Health Interview Survey. BMC Public Health.

[20] Da’ar OB, Alahmary K, Alsalamah M (2019). Association between emergency department visits and predisposing, enabling, need, and reinforcing social factors in an acute care. Athens J Health Medical Sci.

[21] Parashar S, Chan K, Milan D (2014). The impact of unstable housing on emergency department use in a cohort of HIV-positive people in a Canadian setting. AIDS Care.

[22] Fleury MJ, Rochette L, Grenier G, Huỳnh C, Vasiliadis HM, Pelletier É, Lesage A (2019). Factors associated with emergency department use for mental health reasons among low, moderate and high users. Gen Hosp Psychiatry.

[23] Krebs LD, Kirkland SW, Villa-Roel C (2017). Emergency department use: influence of connection to a family physician on ED use and attempts to avoid presentation. Healthc Q.

[24] Cheung PT, Wiler JL, Lowe RA, Ginde AA (2012). National study of barriers to timely primary care and emergency department utilization among Medicaid beneficiaries. Ann Emerg Med.

[25] Guleria I, Campbell JA, Thorgerson A, Bhandari S, Egede LE (2025). Relationship between social risk factors and emergency department use: National Health Interview Survey 2016-2018. West J Emerg Med.

[26] Abbott EE, Taylor S, Vargas-Torres C, Petrozzo K, Buckler DG, Richardson LD, Zebrowski AM (2025). Are unmet health related social needs associated with emergency department utilization among Medicare beneficiaries?. BMC Health Serv Res.

[27] Hong R, Baumann BM, Boudreaux ED (2007). The emergency department for routine healthcare: race/ethnicity, socioeconomic status, and perceptual factors. J Emerg Med.

[28] Shippee ND, Shippee TP, Hess EP, Beebe TJ (2014). An observational study of emergency department utilization among enrollees of Minnesota Health Care Programs: financial and non-financial barriers have different associations. BMC Health Serv Res.

[29] Unger S, Orr Z, Alpert EA, Davidovitch N, Shoham-Vardi I (2022). Social and structural determinants of emergency department use among Arab and Jewish patients in Jerusalem. Int J Equity Health.

[30] Dufour I, Chiu Y, Courteau J, Chouinard MC, Dubuc N, Hudon C (2020). Frequent emergency department use by older adults with ambulatory care sensitive conditions: a population-based cohort study. Geriatr Gerontol Int.

[31] Behr JG, Diaz R (2016). Emergency department frequent utilization for non-emergent presentments: results from a regional urban trauma center study. PLoS One.

[32] Matifary CR, Wachira B, Nyanja N, Kathomi C (2021). Reasons for patients with non-urgent conditions attending the emergency department in Kenya: a qualitative study. Afr J Emerg Med.

[33] Chandekar A, Akande O, Makar C (2026). Impact of primary care visit frequency on non-urgent emergency department visits in a large urban medical center. J Prim Care Community Health.

[34] Afilalo J, Marinovich A, Afilalo M, Colacone A, Léger R, Unger B, Giguère C (2004). Nonurgent emergency department patient characteristics and barriers to primary care. Acad Emerg Med.

[35] Chary A, Bhananker A, Suh M (2025). Drivers of frequent emergency department use in socioeconomically disadvantaged older adults: a qualitative study. J Am Geriatr Soc.

[36] Cho ED, Kim B, Kim DH, Lee SG, Jang SY, Kim TH (2023). Factors related to the frequent use of emergency department services in Korea. BMC Emerg Med.

[37] Zuckerman S, Shen YC (2004). Characteristics of occasional and frequent emergency department users: do insurance coverage and access to care matter?. Med Care.

[38] Petersen LA, Burstin HR, O'Neil AC, Orav EJ, Brennan TA (1998). Nonurgent emergency department visits: the effect of having a regular doctor. Med Care.

[39] Sieck CJ, Hefner JL, Wexler R (2016). Why do they do that?: Looking beyond typical reasons for non-urgent ED use among Medicaid patients. Patient Exp J.

[40] Durand AC, Palazzolo S, Tanti-Hardouin N, Gerbeaux P, Sambuc R, Gentile S (2012). Nonurgent patients in emergency departments: rational or irresponsible consumers? Perceptions of professionals and patients. BMC Res Notes.

[41] Rudge GM, Mohammed MA, Fillingham SC, Girling A, Sidhu K, Stevens AJ (2013). The combined influence of distance and neighbourhood deprivation on emergency department attendance in a large English population: a retrospective database study. PLoS One.

[42] Carlson LC, Kim J, Samuels-Kalow ME, Yun BJ, Terry DF, Weilburg JB, Lee J (2021). Comparing neighborhood-based indices of socioeconomic risk factors and potentially preventable emergency department utilization. Am J Emerg Med.

[43] Ohle R, Bleeker H, Yadav K, Perry JJ (2018). The immigrant effect: factors impacting use of primary and emergency department care - a Canadian population cross-sectional study. CJEM.

[44] Butler PA (1998). Medicaid HMO enrollees in the emergency room: use of nonemergency care. Med Care Res Rev.

[45] McKenna G, Rogers A, Walker S, Pope C (2020). The influence of personal communities in understanding avoidable emergency department attendance: qualitative study. BMC Health Serv Res.

[46] Rust G, Ye J, Baltrus P, Daniels E, Adesunloye B, Fryer GE (2008). Practical barriers to timely primary care access: impact on adult use of emergency department services. Arch Intern Med.

[47] Brown EM, Goel V (1994). Factors related to emergency department use: results from the Ontario health survey 1990. Ann Emerg Med.

